# The effect of surgical weight loss on upper airway fat in obstructive sleep apnoea

**DOI:** 10.1007/s11325-022-02734-8

**Published:** 2022-10-27

**Authors:** Kate Sutherland, Garett Smith, Aimee B. Lowth, Nina Sarkissian, Steven Liebman, Stuart M. Grieve, Peter A. Cistulli

**Affiliations:** 1 Sleep Research Group, Charles Perkins Centre and Sydney Medical School, University of Sydney, Camperdown, NSW 2006 Australia; 2 Department Respiratory & Sleep Medicine, Royal North Shore Hospital, Sydney, NSW Australia; 3 Upper Gastrointestinal Surgical Unit, Royal North Shore Hospital and North Shore Private Hospital, Sydney, NSW Australia; 4 Discipline of Surgery, Northern Clinical School, University of Sydney, Sydney, NSW Australia; 5 Imaging and Phenotyping Laboratory, Charles Perkins Centre and Sydney Medical School, Faculty of Medicine and Health, University of Sydney, and Department of Radiology, Royal Prince Alfred Hospital, Sydney, Australia

**Keywords:** Obstructive sleep apnoea, Magnetic resonance imaging, Weight loss, Upper airway, Tongue fat

## Abstract

**Purpose:**

Obesity is a reversible risk factor for obstructive sleep apnoea (OSA). Weight loss can potentially improve OSA by reducing fat around and within tissues surrounding the upper airway, but imaging studies are limited. Our aim was to study the effects of large amounts of weight loss on the upper airway and volume and fat content of multiple surrounding soft tissues.

**Methods:**

Participants undergoing bariatric surgery were recruited. Magnetic resonance imaging (MRI) was performed at baseline and six-months after surgery. Volumetric analysis of the airway space, tongue, pharyngeal lateral walls, and soft palate were performed as well as calculation of intra-tissue fat content from Dixon imaging sequences.

**Results:**

Among 18 participants (89% women), the group experienced 27.4 ± 4.7% reduction in body weight. Velopharyngeal airway volume increased (large effect; Cohen’s d [95% CI], 0.8 [0.1, 1.4]) and tongue (large effect; Cohen’s d [95% CI], − 1.4 [− 2.1, − 0.7]) and pharyngeal lateral wall (Cohen’s d [95% CI], − 0.7 [− 1.2, − 0.1]) volumes decreased. Intra-tissue fat decreased following weight loss in the tongue, tongue base, lateral walls, and soft palate. There was a greater effect of weight loss on intra-tissue fat than parapharyngeal fat pad volume (medium effect; Cohen’s d [95% CI], − 0.5 [− 1.2, 0.1], *p* = 0.083).

**Conclusion:**

The study showed an increase in velopharyngeal volume, reduction in tongue volume, and reduced intra-tissue fat in multiple upper airway soft tissues following weight loss in OSA. Further studies are needed to assess the effect of these anatomical changes on upper airway function and its relationship to OSA improvement.

**Supplementary Information:**

The online version contains supplementary material available at 10.1007/s11325-022-02734-8.

## Introduction

Obstructive sleep apnoea (OSA) is a common sleep disorder estimated to affect nearly one billion people globally [1]. OSA is characterised by the repetitive collapse of the pharyngeal airway during sleep which impedes airflow resulting in intermittent hypoxia and sleep fragmentation. OSA is associated with daytime symptoms, reduced quality of life, increased accident risk, increased risk of cardiometabolic dysfunction, and disease and all-cause mortality [2–4]. Obesity is the major reversible risk factor for OSA, and therefore weight loss is a treatment strategy for OSA with additional parallel cardiometabolic and quality of life benefits [5].

The effect of weight loss on OSA is highly variable and does not strongly correlate with the amount of weight loss achieved. Large amounts of weight loss resulting from bariatric surgery only ameliorates OSA in 38% of patients [6], but conversely small amounts of weight loss can have a substantial effect on improving OSA in some people [7]. Overall, there is only a modest relationship between total body weight loss and OSA improvement. The effectiveness of weight loss as an OSA therapy may depend on regional changes in adiposity.

Factors outside the upper airway, such as neurohumoral alterations in respiratory drive and lung volume increase secondary to abdominal fat reduction, likely contribute to reducing parameters in OSA  following weight loss [8, 9]. Around the pharynx, shrinking of regional fat deposits may reduce collapsibility from extraluminal tissue pressure [10]. We have previously shown reduction in facial and parapharyngeal fat following weight loss and increased velopharyngeal airway space [11]. Fat deposition is increased in the tongue in OSA, and tongue fat reduction following weight loss specifically explains some of the reduction in OSA severity [12]. To our knowledge, a detailed exploration of parapharyngeal and intra-tissue fat in different upper airway soft tissues following weight loss has not been undertaken.

The overall aim of this exploratory study was to image upper airway regional and intra-tissue fat content in patients with OSA before and after surgical weight loss to advance understanding of the pharyngeal mechanisms of weight loss on OSA improvement. We aimed to quantify regional soft tissue adiposity and airway changes following weight loss.

## Methods

### Participants

Bariatric surgery was selected as the method of weight loss for this exploratory study as large amounts of body weight loss are achieved in a relatively short timeframe (maximum weight loss at around 6 months). The study was approved by the Northern Sydney Local Health District (NSLHD) Human Research Ethics Committee (Protocol numbers HREC/15/HAWKE/386, RESP/15/278). All participants gave written informed consent. Participants were recruited from a private clinic in Sydney, Australia. Exclusion criteria were a known history of syndromal craniofacial abnormalities, previous craniofacial or upper airway surgery, or significant upper airway deformity or obstruction that is not obesity-related (e.g. enlarged tonsils, or nasal obstruction); contra-indications to MRI (incompatible implants, claustrophobia, pregnancy, exceeding scanner size limits [weight > 200 kg, waist circumference > 220 cm]); or dental work which may cause artefact. Participants underwent polysomnography and OSA treatment as required (detail in *online supplement*).

### Magnetic resonance imaging

Magnetic resonance imaging (MRI) was performed at baseline and 6 months post-surgery. Participants were positioned as per previous upper airway imaging protocols [13]. Additional details are provided in the *online supplement*. The MRI protocol was modified Dixon (mDixon) which produces four sets of images, including fat- and water-saturated images which can be utilised for fat quantification [14].

### Upper airway and soft tissue analysis

**Fig. 1:**
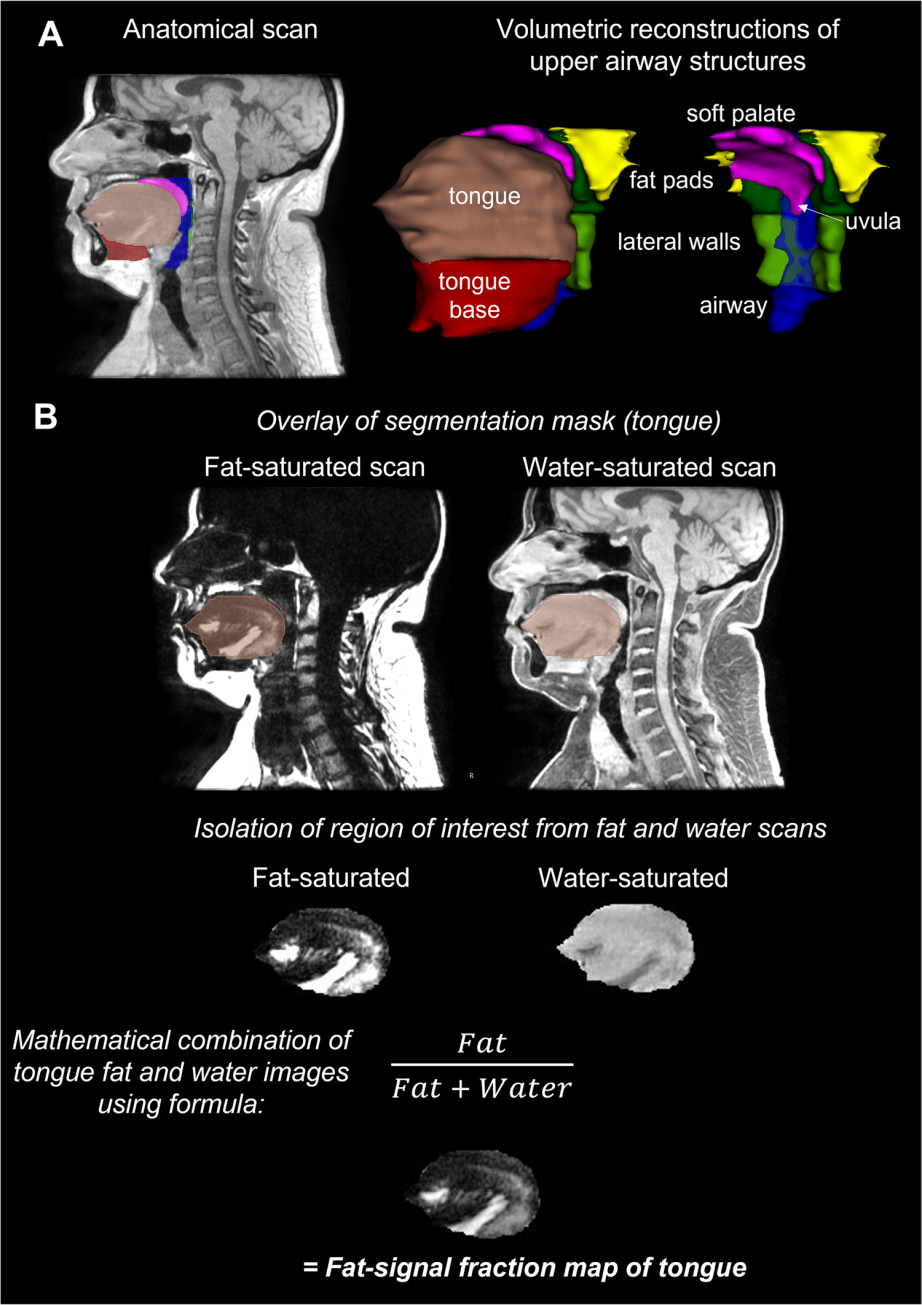
Volumetric analysis of upper airway structures and intra-tissue fat quantification. **A** Segmentation of the borders of upper airway structures of interest are performed slice by slice. Different colour ‘masks’ represent the individual structures. Volumetric reconstructions of each structure are presented. Light red = tongue; dark red = tongue base; pink = soft palate; dark green = velopharyngeal lateral walls; light green = oropharyngeal lateral walls; yellow = parapharyngeal fat pads; blue = airway space. **B** Illustration of the process for intra-tissue fat quantification using example of the upper tongue. The mDixon imaging method produces four scans: in-phase, out-phase, fat-saturated, and water-saturated. The anatomical boundaries are not clear on the fat-weighted images to allow segmentation of specific tissues. Therefore, the segmentation mask from the anatomical scan is overlayed on the fat and water scans to mark the tissue boundaries of interest. All voxels outside of the three-dimensional tissue mask are then deleted, resulting in an image of only the tissue structure of interest. The fat and water scans of the tissues can then be mathematically combined using the displayed equation to produce a fat-signal fraction map of the tissue of interest, in this case the tongue. In the fat-signal fraction map image, the value of each voxel represents the fat percentage within that voxel. Voxels with high fat content appear white, while those with low fat content appear black. The fat percentage of the entire tissue structure can then be determined. The same process was used to obtain fat content of the soft palate and tongue base

Image analysis was performed in 3D slicer software (http://www.slicer.org) [15]. Volumetric analysis of the upper airway space and soft tissues was performed according to previously published protocols [13]. Additional detail can be found in the *online supplement*. Briefly, axial image slices from the anatomical scans were used to segment structures of interest to create volumetric reconstructions of these structures (Fig. 1A). The upper airway was subdivided into regions of the velopharynx (hard palate to uvula tip), oropharynx (uvula tip to epiglottis base), and hypopharynx (epiglottis base to vocal fold) [13]. Soft tissue segmentation included the soft palate; tongue in two sections, the upper tongue (genioglossus muscle and tongue dorsum) and tongue base (including geniohyoid, mylohyoid, hyoglossus muscles); lateral pharyngeal walls divided into two regions, velopharyngeal and oropharyngeal; and parapharyngeal fat pads (regional fat deposits). To quantify intra-tissue fat within each tissue boundary, the segmentation mask was used to identify the structure on both the fat- and water-saturated scans (Fig. 1B). The regions within the mask from both scans are mathematically combined to produce a fat-signal fraction map of that tissue [16, 17]. The signal intensity of each voxel in this resulting image represents the percent of fat in that voxel.

### Statistical analysis

Statistical analysis was performed using IBM SPSS Statistics (Version 26). Changes in anthropometric and upper airway structure measurements following weight loss intervention were assessed using paired *t*-tests. The effect size of these changes was calculated using Cohen’s d for repeated measures (Cohen’s d_rm_) [18]. This was an exploratory analysis to assess a range of anatomical upper airway factors; no adjustment for multiple comparisons was made. Statistical significance was accepted at *P* < 0.05.

## Results

### Participant characteristics and weight loss

**Fig. 2:**
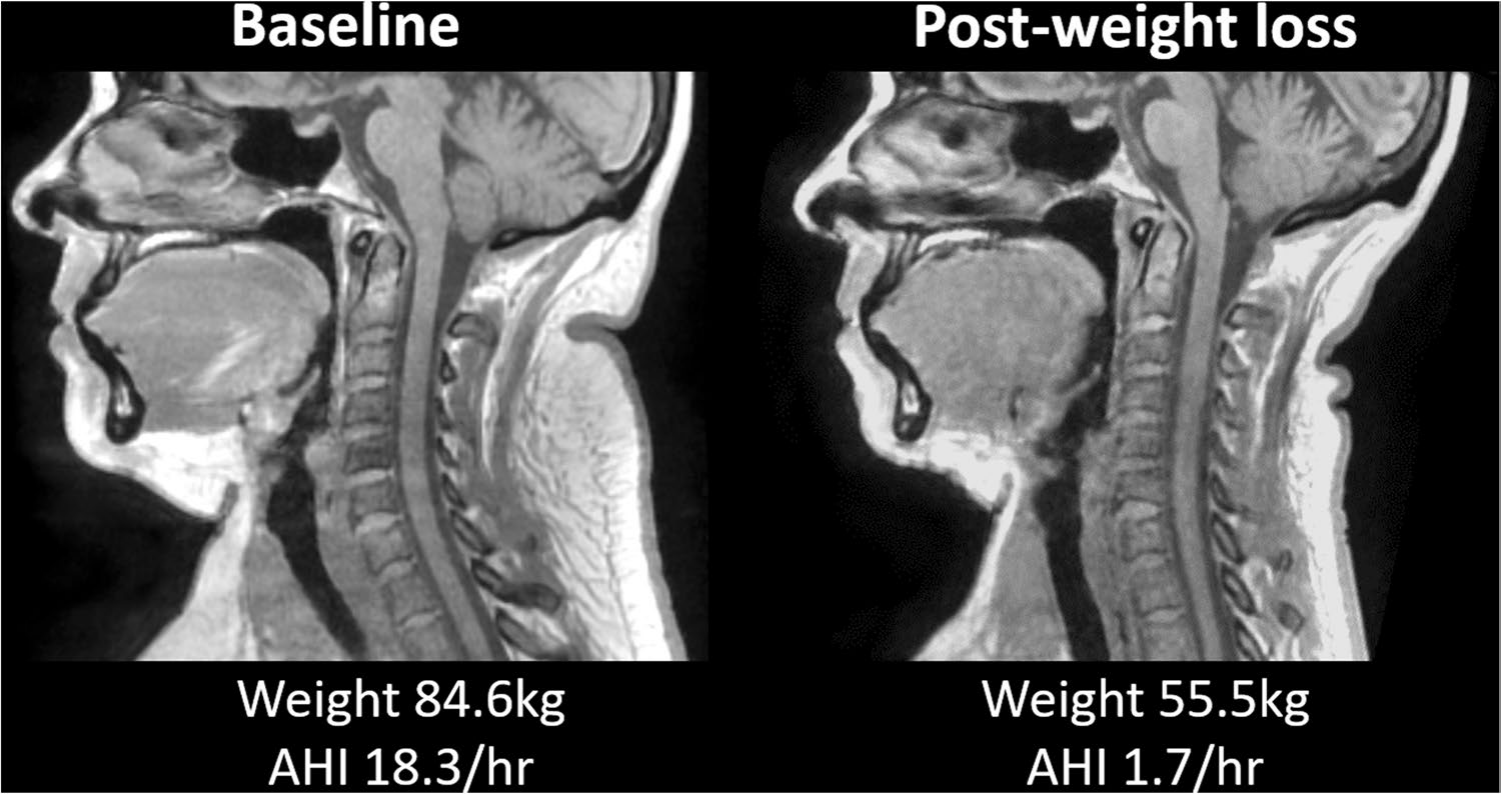
Mid-sagittal scan before and after weight loss surgery. This female participant (50 years) lost 35% of her baseline weight 6 months after surgery and no longer showed evidence of obstructive sleep apnoea with an AHI reduction of 91%. In this example, participant fat loss can be observed in the head and neck regions 6 months after weight loss surgery (in phase, anatomical images). Reduction in adipose tissue (appears white) can be observed clearly in the submental region and back of the neck. An antero-posterior widening of pharyngeal region can be observed in the post-weight loss image and reduction in tongue cross-sectional area

Eighteen participants with OSA were recruited (Table 1). The weight loss surgery performed in the recruited participants was predominantly sleep gastrectomy, but three of the participants underwent a laparoscopic mini bypass procedure. The samples were predominantly female (89%), and all self-reported white ethnicity. In terms of OSA severity, 22% had mild OSA, 44% moderate, and 33% severe. Six months after weight loss surgery, the sample had lost on average > 30 kg (27.4 ± 4.7% body weight reduction) with 12.3% reduction in neck circumference and 18.8% reduction in waist circumference (Table 1). There was on average > 60% reduction in AHI, with 38.9% no longer classified as having OSA (AHI < 5 events/hour). An example mid-sagittal image of an individual participant before and after weight loss is shown in Fig. 2.

**Table 1:** Participant characteristics and effects of bariatric surgery for weight loss. Eighteen participants with obstructive sleep apnoea (AHI > 5 events/hour) were included. Post-weight loss assessment was 6 months after surgery. *P* value is from paired t-test comparison of baseline and post-surgery values, *P < 0.001

	Baseline	Post-weight loss surgery	Absolute change	% Change	*P *value
*Demographics*					
Age (years)	46.1 ± 9.5				
Gender (F/M)	16/2				
* Anthropometry*					
BMI (kgm^2^)	44.1 ± 7.3	32.0 ± 6.0	− 12.1 ± 3.2	− 27.4 ± 6.2	< 0.001*
Weight (kg)	123.1 ± 21.6	90.0 ± 18.5	− 33.4 ± 6.7	− 27.4 ± 4.7	< 0.001*
Neck circumference (cm)	42.4 ± 4.0	37.1 ± 3.3	− 5.3 ± 2.7	− 12.3 ± 5.5	< 0.001*
Waist circumference (cm)	128.1 ± 11.4	104.1 ± 12.3	− 24.1 ± 6.8	− 18.8 ± 5.7	< 0.001*
Hip circumference (cm)	140.2 ± 17.2	117.3 ± 14.9	− 22.9 ± 8.0	− 16.2 ± 5.1	< 0.001*
* Polysomnography*					
AHI (events/hour)	23.6 ± 13.1	7.8 ± 5.7	− 15.8 ± 13.5	− 61.6 ± 38.2	< 0.001*

### Effect of weight loss on upper airway structures and adiposity

**Fig. 3:**
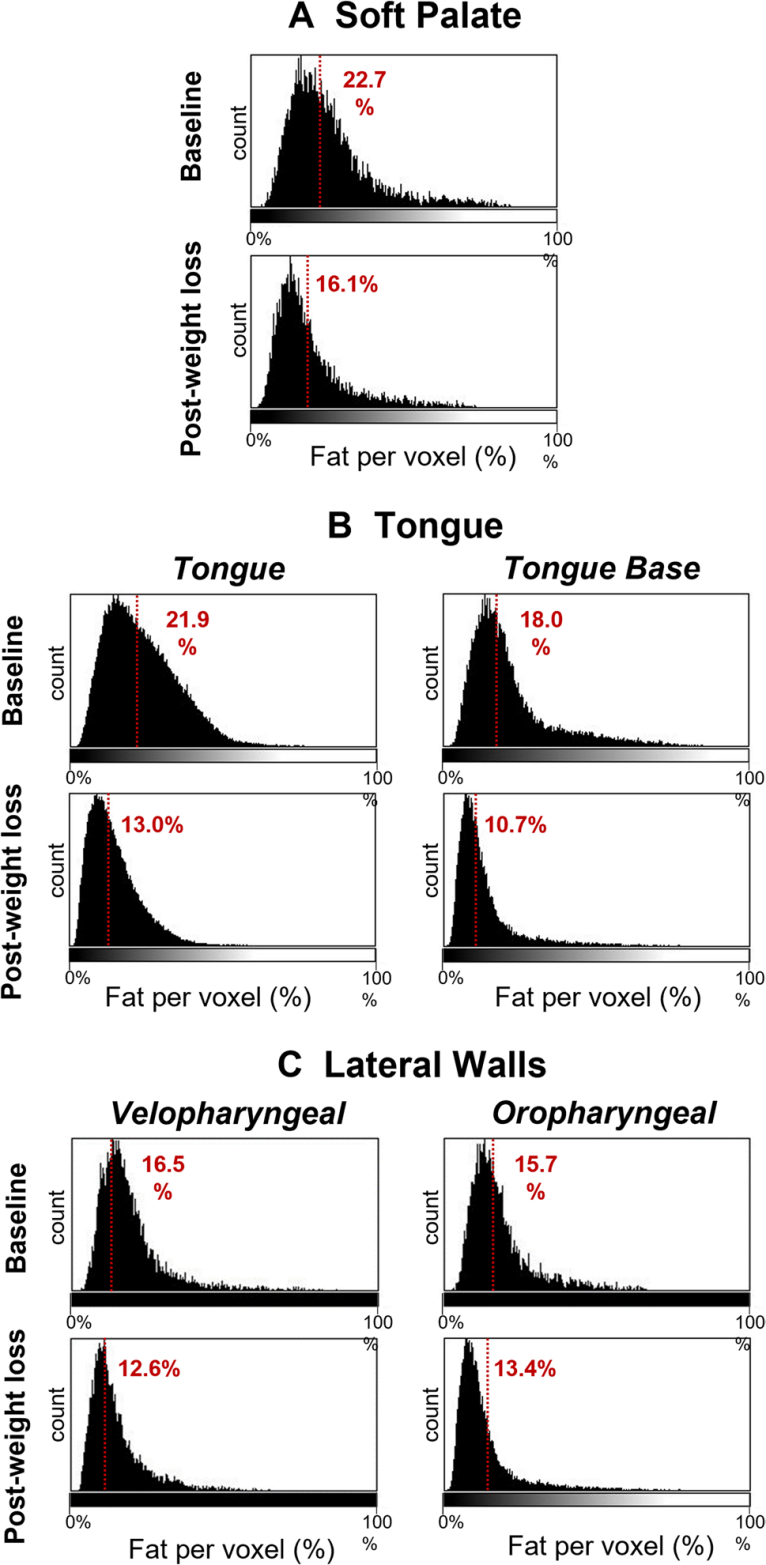
Changes in intra-tissue fat following weight loss. Histograms of voxel fat fraction (0–100%) by tissue type before and after weight loss in a single participant for **A** soft palate, **B** tongue (tongue and tongue base), and **C** pharyngeal lateral walls (velopharyngeal and oropharyngeal regions). The participant is the female shown in Fig. 2. Median fat percentage for each histogram is indicated. The reduction in intra-tissue fat following weight loss can be observed in leftward shifts of the curves in the follow-up histograms

Reproducibility of upper airway measurements is shown in Table S1. Table 2 shows the volumetric changes in the upper airway space, surrounding soft tissues and adiposity. There was an average increase of 2.2cm^3^ in total airway volume. The greatest effect in airway size increase was in the velopharyngeal region. Additional detail of upper airway two-dimensional geometry changes are presented in the *online supplement* (Table S2). In terms of soft tissue structures, both the tongue and tongue base muscles reduced in volume by around 10% (Table 2). However, there was no statistically significant reduction in soft palate volume. The intra-tissue fat content reduced in all upper airway tissue segments; the strongest effect for intra-tissue fat reduction appeared to be in the upper tongue and velopharyngeal lateral wall tissue regions (Cohen’s *d* = 1.6, *P* < 0.001). Regional fat deposition in volume of the parapharyngeal fat pads deceased, although to a lesser extent than intra-tissue fat and was not statistically significant in this sample (Table 2). The reduction in intra-tissue fat content for a single participant are illustrated using histograms of voxel fat percentages by soft tissue type in Fig. 3.

**Table 2:** Changes in upper airway structures and adiposity (regional and intra-tissue fat) following surgical weight loss. Pre and post weight loss regional (parapharyngeal fat pads) and intra-tissue fat of the soft palate and tongue are shown (*N* = 18). Intra-tissue fat is expressed as volume of fat within that tissue type, as well as the percent of fat in total tissue volume (% of volume). Effect size is Cohen’s d (for repeated measures); > 0.2 small effect, > 0.5 medium effect, > 0.8 large effect. *P < 0.05, paired *t*-test

	Baseline	Post-weight loss surgery	Absolute change	% Change	*P* value	Effect size Cohen’s d_*rm*_ (95% CI)
Upper airway space						
Total volume (cm^3^)	12.1 ± 2.9	14.0 ± 3.9	+ 2.2 ± 4.3	+ 21.1 ± 41.4	0.089	0.5 (0.2, 1.2)
Velopharyngeal volume (cm^3^)	4.2 ± 1.4	5.3 ± 1.8	+ 1.1 ± 1.7	+ 36.1 ± 51.1	0.010*	0.8 (0.1, 1.4)
Oropharyngeal volume (cm^3^)	4.7 ± 1.6	5.6 ± 2.7	+ 0.9 ± 3.1	+ 32.4 ± 79.7	0.238	0.4 (− 0.3, 1.1)
Hypopharyngeal volume (cm^3^)	3.3 ± 1.4	3.1 ± 0.9	− 0.1 ± 0.9	+ 4.8 ± 35.7	0.524	− 0.2 (− 0.9, 0.5)
Soft tissue						
Soft palate volume (cm^3^)	9.0 ± 2.5	8.7 ± 2.1	− 0.4 ± 1.4	− 1.9 ± 14.2	0.285	− 0.2 (− 0.9, − 0.4)
Tongue volume (cm^3^)	89.2 ± 18.2	80.2 ± 13.2	− 9.0 ± 7.0	− 9.3 ± 6.5	< 0.001*	− 1.5 (− 2.3, − 0.8)
Tongue base volume (cm^3^)	28.5 ± 6.7	25.3 ± 6.1	− 3.2 ± 2.4	− 11.1 ± 8.9	< 0.001*	-1.3 (− 2.0, − 0.6)
Velopharyngeal lateral walls volume (cm^3^)	11.2 ± 1.4	10.1 ± 1.2	− 1.1 ± 1.5	− 8.7 ± 13.2	0.006	− 0.7 (− 1.2, − 0.1)
Oropharyngeal lateral walls volume (cm^3^)	9.0 ± 2.5	8.6 ± 2.3	0.4 ± 3.2	+ 3.4 ± 42.7	0.578	− 0.1 (− 0.8, 0.6)
Intra-tissue fat content						
Soft palate fat volume (cm^3^)	2.7 ± 0.9	2.2 ± 0.9	− 0.5 ± 0.6	− 18.2 ± 18.3	0.002	− 0.9 (− 1.5, − 0.2)
Soft palate fat (% of volume)	29.6 ± 6.0	24.9 ± 7.1	− 4.8 ± 5.4	-	0.001*	− 1.0 (− 1.7, − 0.3)
Tongue fat volume (cm^3^)	27.1 ± 7.5	20.3 ± 5.7	− 6.8 ± 4.1	− 24.5 ± 13.5	< 0.001*	− 1.6 (− 2.4, − 0.9)
Tongue fat (% of volume)	30.4 ± 6.1	25.4 ± 6.3	− 5.0 ± 3.2	-	< 0.001*	− 1.6 (− 2.3, − 0.8)
Tongue base fat volume (cm^3^)	6.7 ± 3.0	4.0 ± 1.4	− 2.8 ± 2.6	− 36.7 ± 21.4	< 0.001*	− 0.9 (− 1.6, − 0.2)
Tongue base fat (% of volume)	23.4 ± 7.8	15.5 ± 3.4	− 7.8 ± 8.1	-	0.001*	− 0.8 (− 1.4, − 0.1)
Velopharyngeal lateral walls fat volume (cm^3^)	2.1 ± 0.5	1.5 ± 0.2	0.7 ± 0.3	− 28.7 ± 15.1	< 0.001*	− 1.6 (− 2.8, − 1.3)
Velopharyngeal lateral walls fat (% of volume)	0.2 ± 0.03	0.1 ± 0.02	0.04 ± 0.02	-	< 0.001*	− 1.6 (− 2.8, − 1.3)
Oropharyngeal lateral walls fat volume (cm^3^)	1.9 ± 0.7	1.3 ± 0.4	0.6 ± 0.04	+ 2.2 ± 2.2	0.004*	− 0.6 (− 1.2, 0.1)
Oropharyngeal lateral walls fat (% of volume)	0.2 ± 0.03	0.2 ± 0.03	0.06 ± 0.04	-	< 0.001*	0 (− 0.7, 0.7)
Regional fat deposition						
Parapharyngeal fat pads (cm^3^)	6.9 ± 2.0	6.2 ± 2.2	− 0.7 ± 1.7	− 8.9 ± 25.7	0.083	− 0.5 (− 1.2, 0.1)

CI, confidence interval; rm, repeated measures

## Discussion

This detailed imaging analysis assessed regional (parapharyngeal fat pads) and intra-tissue fat in multiple soft tissues surrounding the upper airway (soft palate, pharyngeal lateral walls, tongue, and tongue base muscles) following surgical weight loss in OSA. Surgical weight loss reduced intra-tissue fat (large effect) of the soft palate, lateral walls, tongue, and tongue base muscles, while regional fat in the parapharyngeal fat pads reduced to a lesser extent (medium effect). This imaging assessment extends intra-tissue fat imaging to multiple upper airway soft tissues and provides a basis for anatomical assessment to further understand of the pharyngeal mechanisms of weight loss on OSA improvement.

Although weight loss improves OSA [5], there are minimal studies providing understanding of the mechanisms by which weight loss affects upper airway structure and reduces collapsibility. Excess soft tissue surrounding the upper airway increases extraluminal pressure exerting a collapsing force on the airway. Animal studies show that increasing mass adjacent to the anterolateral airway walls increases tissue pressure with a corresponding decrease in airway cross-sectional area [10]. Fat deposition around the retropalatal airway and particularly in the parapharyngeal fat pads is greater in OSA patients versus controls and associated with a positive, rather than negative, airway closing pressure [19–21]. In OSA patients, the size of the parapharyngeal fat pads has also been shown to influence the pattern of airway collapse in the retropalatal region with greater collapsibility of the lateral rather than anteroposterior sides of the airway [22]. In our sample, the reduction of fat specifically in the parapharyngeal fat pads was less prominent with overall reduction in these fat masses on average < 10% of baseline volume. Other studies have noted a greater decrease in fat pad volume in the order of 20–30% despite overall less weight change due to non-surgical weight loss [11, 12]. It is unclear if this may be due to gender differences between the sample in fat loss distribution. However, our image analysis shows a decrease in total size and intra-tissue fat within the surrounding airway tissues following weight loss. This supports that reduced extraluminal tissue pressure from local soft tissue reduction could contribute to reducing propensity for airway collapse following weight loss. Intra-tissue fat reduction in structures such as the soft palate and tongue could also potentially improve contractility [23]. A recent study specifically looking at intra-tongue fat changes following weight loss found a relationship to OSA improvement (AHI reduction), whereas other upper airway measurement changes (airway size, soft tissue, or fat pad volumes) did not [12], although the fat content of other surrounding upper airway soft tissues was not assessed in the study. Our study was a small exploratory study, and we are likely underpowered to assess relationships between anatomical and AHI changes, as weight loss itself was not significantly correlated with AHI in this sample (online supplement Table S3). Accordingly, we did not find any correlations with upper airway fat changes and AHI change, although we did find a relationship with soft palate and velopharyngeal lateral wall total volume reduction (Table S3).

Reduced upper airway space is an anatomical feature of those with OSA compared to controls [24]. Reduced extraluminal tissue pressure from fat loss may allow expansion of the airway cross-sectional area and reduce resistance and increase airflow in accordance with Poiseuille’s Law of flow through a tube [25]. In this study, we confirm that the greatest effect of weight loss on increasing airway space occurs in the velopharynx [11]. We found an increase in total airway volume in this region as well as in cross-sectional area (online supplement Table S2).

The other anatomical aspect of airway shape affecting resistance to airflow is airway length, with resistance proportional to length in Poiseuille’s Law [25]. Previously, we have shown airway length decrease to be related to AHI reduction in men following medical weight loss [11]. Increased airway length has been implicated as a predominant characteristic explaining greater male predisposition to OSA [26]. Increased lung volume as a result of abdominal fat loss likely plays a large role in the improvement of OSA following weight loss [9]. Increasing lung volume increases pharyngeal cross-sectional area and deceases pharyngeal collapse [27, 28]. Presumably caudal traction on upper airway structures somehow results in a widening of cross-sectional area and reduction in total upper airway length [9]. We did also investigate a measure of airway length in the distance between the first and last image slice in each airway region but did not find a change in airway length following weight loss from image analysis in this sample (online supplement Table S2) with the only notable airway shape change being velopharyngeal volume and cross-sectional area. The relative importance of different mechanisms leading to OSA improvement following weight loss may also vary by sex or depending on individual OSA pathophysiology. To fully understand weight loss mechanisms of OSA improvement, a combination of multiple anatomical changes as well as functional effects need to be assessed concurrently. These data produced in the current study will aid in powering larger studies to look at anatomical effects of weight loss on the upper airway.

To the best of our knowledge, this is the first study to quantify intra-tissue fat in multiple upper airway soft tissue structures (soft palate, upper and lower tongue, and pharyngeal lateral walls) following surgical weight loss. We show reduction in intra-tissue fat in all of these tissues. This shows the feasibility of exploring the contribution of areas of adiposity to airway collapse and strong reproducibility of these measurements in weight loss images.

However, there are some important limitations. The sample size for this exploratory study was modest but was nevertheless able to detect significant changes as a result of the large effects associated with major weight loss. All participants achieved large weight loss through bariatric surgery; therefore, we have a relatively narrow range of weight loss which may additionally affect our ability to detect relationships with other variables over a larger spread of weight loss amounts. The bariatric surgery recruitment in our study resulted in a predominantly female sample. There are noted differences in fat distribution between men and women in OSA [29], and the findings may differ in a male sample. The current sample is not sufficient to allow stratification to look at weight loss effects on upper airway fat in relation to sex or menopausal status in women. Larger studies would be needed to understand any role of these factors in the effect of weight loss on upper airway fat distribution and corresponding effects on OSA severity. This study has the limitations of all awake imaging studies in that airway structure changes with sleep onset, and therefore direct relationship to sleep parameters is reduced. However, sleep state should not affect the fat content within upper airway tissues. Although head position was standardised for the scan, the weight loss reduced neck fat which may result in differences in neck position relative to head position between scans performed on separate occasions which could affect airway space measurements. Upper airway anatomical changes are just one mechanism which may improve OSA following weight loss intervention, and we did not assess changes in lung volume or neurohumoral effects on respiratory drive as other potential contributing mechanisms [9]. Future studies assessing different mechanisms concurrently and the functional effects of upper airway anatomical changes are warranted.

## Conclusion

Weight loss resulting from bariatric surgery increases upper airway space in the velopharynx and reduces soft tissue volumes and intra-tissue fat content with a large effect size. This is the first assessment of intra-tissue fat in multiple soft tissues around the airway (tongue, soft palate, pharyngeal lateral walls).

## Supplementary Information

Below is the link to the electronic supplementary material.

Supplementary file1 (DOCX 57 KB)

## Data Availability

The datasets generated during and/or analysed during the current study are not publicly available due to privacy reasons but are available from the corresponding author on reasonable request.
