# Supplementary material for: The effect of surgical weight loss on upper airway fat in obstructive sleep apnoea
Source: Sleep Breath. 2022 Oct 27;27(4):1333–41. doi: 10.1007/s11325-022-02734-8 (PMC10427513; doi:10.1007/s11325-022-02734-8)
Supplement: Supplementary file 1 — Supplementary file1 (DOCX 57 KB) [file 11325_2022_2734_MOESM1_ESM.docx]

ONLINE SUPPLEMENT

**The effect of surgical weight loss on** **upper airway fat in Obstructive Sleep Apnoea**

Kate Sutherland^1,2^, Garrett Smith^3,4^, Aimee B Lowth^1,2^, Nina Sarkissian^1,2^, Steven Liebman^3^, Stuart M Grieve^5^, Peter A Cistulli^1,2^

*^1^Sleep Research Group, Charles Perkins Centre and Sydney Medical School, University of Sydney, NSW, Australia; ^2^Department Respiratory & Sleep Medicine, Royal North Shore Hospital, Sydney, NSW, Australia; ^3^Upper Gastrointestinal Surgical Unit, Royal North Shore Hospital and North Shore Private Hospital, Sydney, NSW, Australia; ^4^Discipline of Surgery, Northern Clinical School, University of Sydney, NSW, Australia; ^5^Imaging and Phenotyping Laboratory, Charles Perkins Centre and Sydney Medical School, Faculty of Medicine and Health, University of Sydney, and Department of Radiology, Royal Prince Alfred Hospital, Sydney, Australia.*

**METHODS**

*Bariatric surgery*

The majority of participants underwent sleeve gastrectomy surgery for weight loss, but three underwent a laparoscopic mini bypass procedure.

*Polysomnography*

At study entry all participants underwent overnight polysomnography in the clinical sleep laboratory of Royal North Shore Hospital. Polysomnography (Compumedics, Melbourne, Australia) was performed according to standard procedures with channels for electroencephalogram (EEG), electro-oculogram (EOG), chin electromyogram (EMG), electrocardiogram (ECG), anterior tibial EMG, nasal pressure, chest and abdomen movements, body position and arterial oxygen saturation (SpO_2_). All studies were scored by experienced scorers in accordance with the 2012 American Academy of Sleep Medicine (AASM) scoring manual. [1] Specifically, hypopneas were scored if the airflow signal reduced by ≥30% of baseline with ≥3% oxygen desaturation or cortical arousal. Participants who were identified to have OSA at baseline (AHI ≥ 5 events/hour) were included in this analysis and repeated polysomnography 6 months post-surgery. The polysomnography and MRI scan were performed within one week of each other at both time points (and in most cases were on consecutive days).

*OSA Treatment*

Participants diagnosed with OSA were reviewed by a board-certified sleep physician to decide whether specific therapy was required while waiting to assess the effect of weight loss. Where treatment was recommended, it was in the form of automatic positive airway pressure (APAP) on the basis that treatment pressures would automatically adjust in the event of OSA improvement resulting from weight loss. In such patients APAP was withheld for 1 week prior to the post-surgery sleep study in order to avoid a potential carryover effect.

*Magnetic resonance imaging*

Magnetic resonance imaging (MRI) was performed at baseline and 6 months post-surgery. Participants were in the supine position on the MRI scanner bed with the head position standardised to position the Frankfort plane perpendicular to the scanner bed and secured with foam wedges, according to previous protocols. [2, 3] Participants were instructed to have their lips and teeth lightly touching with the tip of the tongue resting on the back of the front teeth and to breath quietly through their nose for duration of the scan.

Modifed Dixon (mDixon) imaging utilises the chemical shift difference between water and fat to produce in-phase and out-of-phase images which can be used to generate separate fat-saturated and water-saturated images [4] which allows quantification of fat content within tissues. mDixon images were acquired using a 3.0 Tesla Discovery MR 750w scanner (GE Medical systems) at Macquarie Medical Imaging (Macquarie Park, Sydney, Australia). The image slice thickness was 1mm isotropic.

*Upper airway and soft tissue analysis*

Image analysis was performed in 3D slicer software (<http://www.slicer.org>). [5] The upper airway was analysed as volumes of the airway space and upper airway soft tissues according to previously published methods. [2, 3, 6] Segmentations of upper airway structures of interest were performed by a researcher experienced in upper airway analysis protocols utilising manual and semi-automated segmentation tools of the program. The in-phase image allows adequate visualisation of the upper airway soft tissue borders and the axial image slices were used for these anatomical segmentations (**Figure 1A**). The upper airway space was segmented from the hard palate to above the vocal folds using the level tracing tool of the software (defines an outline where pixels all have the same background value as the current background pixel). The airway volume was subdivided into regions of the velopharynx (hard palate to uvula tip), oropharynx (uvula tip to epiglottis base) and hypopharynx (epiglottis base to vocal fold). [2] The airway length was taken as the number of slices between these landmarks. The cross-sectional area (CSA) on each axial image slice was also obtained, and average (mean), minimum and maximum CSA for each airway region determined. A threshold tool set to capture fat was used to segment the structure of the parapharyngeal fat pads. Manual segmentation (paint brush tool) was used to define the boundaries of the tongue (genioglossus muscle and tongue dorsum) and soft palate (**Figure 1A**). The base of the tongue was segmented separately. This region contains the sublingual salivary glands and other tongue muscles (geniohyoid, mylohyoid, hyoglossus) which are difficult to separate anatomically but contribute tissue bulk to the oral cavity space. The lateral pharyngeal walls were segemented to include the pharyngeal mucosal space and the retropharyngeal space which was not included in other segmented structures (soft palate, tongue, parapharyngeal fat pads, airway space). The lateral walls were segmented from the hard palate superiorly to the base of the epiglottis inferiorly. The lateral walls were further divided based on the airway region into the velopharyngeal lateral walls (from hard palate to uvula tip) and oropharyngeal lateral walls (from uvula tip to epiglottis base). The right masseter muscle was additionally segmented from the level of the hard palate until it disappeared caudally. This structure was selected as a control tissue volume as it sits outside the mandibular bone (not presented in this analysis). The masseter volume would not contribute to extraluminal tissue pressure on the pharyngeal airway and therefore could be a potential tissue structure to help distinguish direct tissue effects on the pharyngeal airway compared to generalised associations with weight loss. The axial segmentations produce 3D reconstructions of the tissues and volume data (**Figure 1A**).

Although blinding of the scan sequence (i.e. before and after surgery) was attempted, in reality this is difficult due to the study sample being obese at baseline and losing a large amount of weight on the second scan which is visually discernible. An important part of this study was to assess the reproducibility of the upper airway tissue measurements in order to evaluate validity of assessing volume changes between scans on separate occasions. Therefore, each scan was analysed on three separate occasions (>1 month between each analysis) to assess measurement reproducibility. The average volume for each scan was used in analysis.

*Intra-tissue fat quantification*

The process for intra-tissue fat quantification is illustrated for tongue tissue in **Figure 1B**. The fat-saturated and water-saturated images generated from the mDixon imaging sequence can be used to generate a fat-signal fraction image map. [7, 8] A fat-signal fraction map is produced from the signal intensities of the voxels in the water and fat scans which can be used to calculate the fat within each voxel according to the formula: [7]

$$fat signal fraction= \frac{{SI}_{fat}}{{SI}_{fat}+{SI}_{water}} X 100\%$$

Where SI_fat_ is the signal intensity of the fat-saturated image and SI_water_ is the signal intensity of the water-saturated image. First the segmentation of the tissue of interest (obtained from the anatomical image, described above) was used as a mask to identify the tissue boundaries on the fat and water images (**Figure 1B**). The voxels outside the tissue mask was deleted (using the Mask Scaler Volume module of 3D slicer) to create a fat-saturated and water-saturated image of the tissue of interest. These fat and water images were combined according to the above formula using the Math functions in imaging software Image J (ImageJ 1.52a, National Institutes of Health, USA). The resulting image is the fat-signal fraction map of the tissue. The fat percentage of the tissue was calculated as the average fat fraction of all voxels within that volume.

*Statistical analysis*

Repeatability of upper airway segmentation measurements were assessed using Intra-class correlation coefficients (two-way mixed, absolute agreement). The relationship between changes in upper airway and polysomnographic variables was assessed using correlation analysis. Variables were assessed for normality of distribution (Shapiro-Wilk test) and Pearson’s or Spearman’s rank correlation procedures were used as appropriate.

**RESULTS**

*Repeatability of upper airway measurements*

Repeatability of upper airway measurements is shown in the online supplement (**Table S1)**. The overwhelming majority of measurements on both the pre- and post-weight loss scans showed an ICC value >0.9, indicating excellent intra-rater reliability [9]. The few measurements not achieving ICC >0.9 were still in the good reliability category (ICC 0.75 – 0.9). Therefore, measurement reliability was confirmed for before and after weight loss comparisons.

**Table S1. Repeatability of Upper Airway Measurements**. All upper airway measurements were assessed for intra-rater reliability using the Intra-class correlation procedure (ICC, two-way mixed, absolute agreement). Airway length refers to the number of axial slices counted for each airway region to assess for any differences identification of the landmarks used to designated airway regional boundaries. Baseline and Post weight loss scans were assessed separately for reliability. The interpretation of ICC values is that values less than 0.5 indicate poor reliability, between 0.5 and 0.75 indicate moderate reliability, between 0.75 and 0.9 indicate good reliability, and values greater than 0.9 indicate excellent reliability ^26^.

|  | **Baseline scans** | **Post weight loss scan** |
| --- | --- | --- |
| Measurement | ICC (95% CI) | ICC (95% CI) |
| *Airway volume (cm^3^)* |  |  |
| **Total** | 0.958 (0.912, 0.982) | 0.958 (0.912, 0.982) |
| **Velopharynx** | 0.955 (0.906, 0.981) | 0.959 (0.913, 0.982) |
| **Oropharynx** | 0.950 (0.894, 0.978) | 0.961 (0.919, 0.983) |
| **Hypopharynx** | 0.951 (0.910, 0.982) | 0.854 (0.690, 0.958) |
| *Soft Tissue Volume (cm^3^)* |  |  |
| **Tongue** | 0.973 (0.926, 0.990) | 0.894 (0.775, 0.955) |
| **Tongue base** | 0.926 (0.846, 0.968) | 0.923 (0.805, 0.970) |
| **Soft Palate** | 0.964 (0.922, 0.985) | 0.913 (0.815, 0.963) |
| **Velopharygneal lateral walls** | 0.856 (0.700, 0.945) | 0.925 (0.837, 0.970) |
| **Oropharyngeal lateral walls** | 0.864 (0.700, 0.945) | 0.932 (0.852, 0.973) |
| **Parapharyngeal Fat Pad** (left) | 0.885 (0.756, 0.951) | 0.941 (0.877, 0.975) |
| **Parapharyngeal Fat Pad** (right) | 0.915 (0.821, 0.964) | 0.952 (0.899, 0.980) |
| **Parapharyngeal Fat Pads** (total) | 0.920 (0.831, 0.966) | 0.953 (0.901, 0.980) |

*Changes in upper airway geometry*

Changes in upper airway length, volume and cross-sectional area by region are shown in **Table S2**.

**Table S2. Changes in the Upper Airway space following surgical weight loss**. Pre and post weight loss dimensions of the upper airway space are shown (N=18). Effect size is Cohen’s d (for repeated measures); >0.2 small effect, >0.5 medium effect, >0.8 large effect. *P<0.05, paired t-test.

|  | **Baseline** | **Post-weight loss surgery** | **Absolute Change** | **P value** | **Effect size**  Cohen’s d*_rm_* (95% CI) |
| --- | --- | --- | --- | --- | --- |
| *Total Upper Airway* |  |  |  |  |  |
| **Length** (cm) | 79.0 ± 10.2 | 79.5 ± 11.2 | +1.4 ± 6.2 | 0.357 | 0.2  (-0.4, 0.9) |
| **Cross-Sectional Area** (cm^2^) |  |  |  |  |  |
| Mean | 1.6 ± 0.2 | 1.7 ± 0.4 | +0.2 ± 0.5 | 0.250 | 0.2  (-0.4, 0.9) |
| Minimum | 0.5 ± 0.2 | 0.5 ± 0.3 | +0.1 ± 0.3 | 0.392 | 0  (-0.6, 0.6) |
| Maximum | 3.8 ± 0.7 | 3.9 ± 1.1 | +0.3 ± 1.2 | 0.694 | 0.1  (-0.5, 0.8) |
| **Volume** (cm^3^) | 12.1 ± 2.9 | 14.0 ± 3.9 | +2.2 ± 4.3 | 0.089 | 0.5  (0.2, 1.2) |
| *Velopharynx* |  |  |  |  |  |
| **Length** (cm) | 33.4 ± 3.8 | 32.7 ± 6.2 | -0.7 ± 4.8 | 0.572 | -0.2  (-0.4, 0.9) |
| **Cross-Sectional Area** (cm^2^) |  |  |  |  |  |
| Mean | 1.2 ± 0.4 | 1.6 ± 0.5 | +0.4 ± 0.7 | 0.018* | 1.0  (0.3, 1.7) |
| Minimum | 0.5 ± 0.3 | 0.7 ± 0.5 | +0.2 ± 0.4 | 0.108 | 0.7  (-0.003, 1.3) |
| Maximum | 3.1 ± 1.0 | 3.7 ± 1.1 | +0.8 ± 1.2 | 0.03* | 0.6  (-0.1, 1.3) |
| **Volume** (cm^3^) | 4.2 ± 1.4 | 5.3 ± 1.8 | +1.1 ± 1.7 | 0.010* | 0.8  (0.1, 1.4) |
| *Oropharynx* |  |  |  |  |  |
| **Length** (cm) | 28.2 ± 7.3 | 30.4 ± 7.9 | +2.2 ± 7.6 | 0.228 | 0.3  (-0.4, 1.0) |
| **Cross-Sectional Area** (cm^2^) |  |  |  |  |  |
| Mean | 1.8 ± 0.5 | 1.8 ± 0.7 | +0.02 ± 0.7 | 0.918 | 0  (-0.6, 0.6) |
| Minimum | 1.0 ± 0.5 | 1.0 ± 0.6 | -0.01 ± 0.7 | 0.944 | 0  (-0.6, 0.6) |
| Maximum | 2.8 ± 0.7 | 2.8 ± 1.0 | +0.02 ± 1.0 | 0.946 | 0  (-0.6, 0.6) |
| **Volume** (cm^3^) | 4.7 ± 1.6 | 5.6 ± 2.7 | +0.9 ± 3.1 | 0.238 | 0.4  (-0.3, 1.1) |
|  | **Baseline** | **Post-weight loss surgery** | **Absolute Change** | **P value** | **Effect size**  Cohen’s d*_rm_* (95% CI) |
| *Hypopharynx* |  |  |  |  |  |
| **Length** (cm) | 17.5 ± 3.9 | 17.3 ± 3.3 | -0.2 ± 3.2 | 0.783 | -0.1  (-0.7, 0.6) |
| **Cross-Sectional Area** (cm^2^) |  |  |  |  |  |
| Mean | 1.9 ± 0.7 | 1.8 ± 0.5 | -0.07 ± 0.6 | 0.603 | -0.1  (-0.8, 0.5) |
| Minimum | 0.8 ± 0.4 | 0.9 ± 0.4 | +0.1 ± 0.4 | 0.310 | 0.3  (0.4, 0.9) |
| Maximum | 3.1 ± 1.0 | 2.9 ± 0.7 | -0.2 ± 0.9 | 0.4371 | -0.2  (-0.9, 0.5) |
| **Volume** (cm^3^) | 3.3 ± 1.4 | 3.1 ± 0.9 | -0.1 ± 0.9 | 0.524 | -0.2  (-0.9, 0.5) |

*Relationship of anatomical changes to AHI change following weight loss*

The relationship between AHI changes and anatomical changes are shown in **Table S3**. AHI and soft tissue volume changes were moderately correlated with soft palate volume reduction associated with decreased AHI (r = 0.5, p = 0.027). There was no relationship between changes in upper airway space volume, in total or by regional, and AHI changes. The volume of fat within each tissue (tongue, tongue base, soft palate) or the parapharyngeal fat pads did not relate to AHI changes, expressed either as total fat volume or as a percent of total tissue volume.

**Table S3. Relationship between anatomical changes following surgical weight loss and improvement in Obstructive Sleep Apnoea (OSA) severity**. Anatomical changes included anthropometry measures and changes upper airway space and soft tissues (including intra-tissue fat). Total soft tissue volume includes upper and lower tongue, lateral walls, soft palate and parapharyngeal fat pad volumes. OSA severity was assessed by change in total Apnoea Hypopnea Index (AHI). Pearson’s or Spearman’s rank (^) correlation procedures were used, as appropriate, to assess the relationship between absolute changes in anatomic variables and OSA severity (N=18). *P<0.05, significant correlations are also presented in bold text.

|  | **Δ AHI** | |
| --- | --- | --- |
|  | *r* | *p* |
| Δ Anatomy |  |  |
| *Anthropometry* |  |  |
| **Weight** (kg) | 0.5 | 0.058 |
| **Neck circumference** (cm) | 0.4 | 0.089 |
| **Waist circumference** (cm) | 0.3 | 0.2 |
| *Upper Airway Space* |  |  |
| **Total Length** (cm) | 0.2 | 0.4 |
| **Total Mean CSA** (cm^2^) | 0.1 | 0.7 |
| **Total Minimum CSA** (cm^2^) | -0.2 | 0.4 |
| **Total Maximum CSA** (cm^2^) | 0.4 | 0.1 |
| **Total volume** (cm^3^) | 0.1 | 0.6 |
| **Velopharyngeal Length** (cm) | 0.4 | 0.1 |
| **Velopharyngeal Mean CSA** (cm^2^) | -0.04 | 0.9 |
| **Velopharyngeal Minimum CSA** (cm^2^) | -0.02 | 0.9 |
| **Velopharyngeal Maximum CSA** (cm^2^) | -0.1 | 0.7 |
| **Velopharyngeal volume** (cm^3^) | 0.2 | 0.4 |
| **Oropharyngeal Length** (cm) | -0.1 | 0.7 |
| **Oropharyngeal Mean CSA** (cm^2^) | 0.1 | 0.6 |
| **Oropharyngeal Minimum CSA** (cm^2^) | -0.1 | 0.7 |
| **Oropharyngeal Maximum CSA** (cm^2^) | 0.2 | 0.4 |
| **Oropharyngeal volume** (cm^3^) | 0.04 | 0.9 |
| **Oropharyngeal Length** (cm) | -0.02 | 0.9 |
| **Hypopharyngeal Mean CSA** (cm^2^) | 0.1 | 0.6 |
| **Hypopharyngeal Minimum CSA** (cm^2^) | -0.2 | 0.4 |
| **Hypopharyngeal Maximum CSA** (cm^2^) | 0.3 | 0.2 |
| **Hypopharyngeal volume** (cm^3^) | 0.1 | 0.8 |
| *Upper Airway Soft Tissue* |  |  |
| **Tongue volume** (cm^3^) | 0.1 | 0.6 |
| **Tongue Fat volume** (cm^3^) | 0.1 | 0.6 |
| **Tongue Fat percentage** (%) | 0.1 | 0.7 |
| **Tongue Base volume** (cm^3^) | 0.04 | 0.9 |
| **Tongue Base Fat volume** (cm^3^) ^ | 0.2 | 0.3 |
| **Tongue Base Fat percentage** (%) ^ | 0.1 | 0.6 |
| **Soft Palate volume** (cm^3^) ^ | **0.5** | **0.027*** |
| **Soft Palate Fat volume** (cm^3^) | 0.2 | 0.5 |
| **Soft Palate Fat percentage** (%) | -0.3 | 0.3 |
| **Velopharyngeal Lateral Walls volume** (cm^3^) | **0.5** | **0.020*** |
| **Velopharyngeal Lateral Walls Fat volume** (cm^3^) | 0.3 | 0.169 |
| **Velopharyngeal Lateral Walls Fat percentage** (%) | -0.1 | 0.790 |
|  | **Δ AHI** | |
|  | *r* | *p* |
| Δ Anatomy |  |  |
| **Oropharyngeal Lateral Walls volume** (cm^3^) | 0.1 | 0.702 |
| **Oropharyngeal Lateral Walls Fat volume** (cm^3^) | 0.2 | 0.433 |
| **Oropharyngeal Lateral Walls Fat percentage** (%) | 0.2 | 0.485 |
| **Parapharyngeal Fat Pads volume** (cm^3^) | -0.01 | 1.0 |
| **Total Soft Tissue Volume** (cm^3^) | 0.3 | 0.226 |

AHI – Apnoea Hypopnea Index, CSA – Cross-Sectional Area

**REFERENCES**

1. Berry RB, Budhiraja R, Gottlieb DJ, Gozal D, Iber C, Kapur VK et al. Rules for scoring respiratory events in sleep: update of the 2007 AASM Manual for the Scoring of Sleep and Associated Events. Deliberations of the Sleep Apnea Definitions Task Force of the American Academy of Sleep Medicine. J Clin Sleep Med. 2012;8(5):597-619. doi:10.5664/jcsm.2172.

2. Chan AS, Sutherland K, Schwab RJ, Zeng B, Petocz P, Lee RW et al. The effect of mandibular advancement on upper airway structure in obstructive sleep apnoea. Thorax. 2010;65(8):726-32. doi:10.1136/thx.2009.131094.

3. Schwab RJ, Pasirstein M, Pierson R, Mackley A, Hachadoorian R, Arens R et al. Identification of upper airway anatomic risk factors for obstructive sleep apnea with volumetric magnetic resonance imaging. Am J Respir Crit Care Med. 2003;168(5):522-30. doi:10.1164/rccm.200208-866OC.

4. Ma J. Dixon techniques for water and fat imaging. J Magn Reson Imaging. 2008;28(3):543-58. doi:10.1002/jmri.21492.

5. Fedorov A, Beichel R, Kalpathy-Cramer J, Finet J, Fillion-Robin JC, Pujol S et al. 3D Slicer as an image computing platform for the Quantitative Imaging Network. Magn Reson Imaging. 2012;30(9):1323-41. doi:10.1016/j.mri.2012.05.001.

6. O'Donoghue F J, Meaklim H, Bilston L, Hatt A, Connelly A, Jackson G et al. Magnetic resonance imaging of the upper airway in patients with quadriplegia and obstructive sleep apnea. J Sleep Res. 2018;27(4):e12616. doi:10.1111/jsr.12616.

7. D'Souza A, Bolsterlee B, Herbert RD. Intramuscular Fat in the Medial Gastrocnemius Muscle of People Who Have Had a Stroke. Front Bioeng Biotechnol. 2020;8:613. doi:10.3389/fbioe.2020.00613.

8. Noble JJ, Keevil SF, Totman J, Charles-Edwards GD. In vitro and in vivo comparison of two-, three- and four-point Dixon techniques for clinical intramuscular fat quantification at 3 T. Br J Radiol. 2014;87(1036):20130761. doi:10.1259/bjr.20130761.

9. Koo TK, Li MY. A Guideline of Selecting and Reporting Intraclass Correlation Coefficients for Reliability Research. J Chiropr Med. 2016;15(2):155-63. doi:10.1016/j.jcm.2016.02.012.
